# Diagnostic and Severity Assessment of Acute Pancreatitis Using Acoustic Radiation Force Impulse Shear Wave Elastography (ARFI-SWE): A Case-Control Study

**DOI:** 10.7759/cureus.117113

**Published:** 2026-09-28

**Authors:** Aagam Jain, Ankur Malhotra, Shruti Chandak, Deepti Arora

**Affiliations:** 1 Radiology, Teerthanker Mahaveer Medical College & Research Centre, Moradabad, IND; 2 Pathology, Teerthanker Mahaveer Medical College & Research Centre, Moradabad, IND

**Keywords:** acoustic radiation force impulse, acute pancreatitis, modified ct severity index, pancreatic stiffness, shear wave elastography

## Abstract

Background

Acute pancreatitis has a variable clinical course, making early severity assessment important. Conventional ultrasonography may have limitations, while CT involves radiation exposure. Acoustic radiation force impulse shear wave elastography (ARFI-SWE) provides quantitative assessment of tissue stiffness and may help evaluate pancreatic involvement and disease severity. This study assessed pancreatic ARFI-SWE parameters in acute pancreatitis and their relationship with the modified CT severity index (MCTSI), Revised Atlanta Classification (RAC), and symptom duration.

Methods

This hospital-based case-control study included 46 patients with acute pancreatitis and 46 healthy controls. ARFI-SWE was performed using a Siemens ACUSON Sequoia ultrasound system with a 6C1 HD convex transducer (Siemens Healthineers, Erlangen, Germany). Three measurements were obtained from the pancreatic head and body, with the median value used for analysis. Shear wave velocity was recorded in m/s and stiffness in kPa. Contrast-enhanced CT was performed using a 160-slice UCT780 scanner (United Imaging, Shanghai, China). MCTSI scores were categorized as mild, moderate, or severe. RAC severity was also recorded. Time since symptom onset was categorized as <24 hours, 24-48 hours, and >48 hours.

Results

Age and sex distributions were comparable between patients and controls (age: 44.13 ± 15.71 vs. 38.61 ± 13.21 years, P=0.071; sex, P=0.834). Pancreatic head and body thickness and main pancreatic duct diameter were significantly greater in patients than controls (30.28 ± 3.81 vs. 25.78 ± 2.85 mm; 24.35 ± 3.14 vs. 15.24 ± 2.14 mm; and 3.226 ± 0.520 vs. 1.243 ± 0.513 mm; all P<0.001). Acoustic radiation force impulse (ARFI) head and body velocities were also higher in patients (2.845 ± 0.476 vs. 1.219 ± 0.143 m/s and 2.949 ± 0.642 vs. 1.161 ± 0.139 m/s), as were head and body stiffness values (22.861 ± 5.947 vs. 4.596 ± 0.731 kPa and 22.730 ± 6.040 vs. 4.139 ± 0.639 kPa; all P<0.001). By MCTSI, 15 (32.6%) patients had mild, 21 (45.7%) moderate, and 10 (21.7%) severe disease; by RAC, 13 (28.3%) were mild, 22 (47.8%) moderately severe, and 11 (23.9%) severe. MCTSI and RAC showed a strong association (Fisher-Freeman-Halton exact test=64.516, P<0.001). ARFI parameters increased significantly across MCTSI severity categories (all P<0.001). MCTSI score showed strong correlations with head velocity (ρ=0.839), body velocity (ρ=0.898), head stiffness (ρ=0.805), and body stiffness (ρ=0.811). In regression analysis, body velocity had the highest explanatory value (R²=0.786). For severe MCTSI-defined disease, area under the curve (AUCs) were 0.935 for head velocity, 1.000 for body velocity, 0.854 for head stiffness, and 0.858 for body stiffness. ARFI parameters also increased significantly with increasing time since symptom onset.

Conclusion

ARFI-SWE showed significantly higher pancreatic shear wave velocity and stiffness in patients with acute pancreatitis than in healthy controls. ARFI parameters were strongly associated with MCTSI severity and showed good to excellent discrimination of severe disease, with body velocity demonstrating the strongest association. Increasing values with symptom duration further support ARFI-SWE as a noninvasive adjunct for quantitative assessment of acute pancreatitis severity.

## Introduction

Acute pancreatitis (AP) is a common inflammatory disorder of the pancreas in which early assessment of severity is essential for appropriate management. Its reported global incidence ranges from 4.9 to 73.4 cases per 100,000 individuals. Although most patients have a mild, self-limiting course, severe AP can lead to local and systemic complications, with mortality reported at 20%-40% in severe disease [1]. Early identification of patients at risk of severe disease is therefore important for timely monitoring and treatment [2].

The diagnosis of AP is usually established when at least two of three criteria are present: characteristic upper abdominal pain, elevated pancreatic enzymes, and imaging findings consistent with AP [3]. Serum amylase and lipase are commonly used, but enzyme levels may be normal in approximately 20% of cases, particularly in delayed presentation, hypertriglyceridemia-related, alcoholic, chronic, or relapsing pancreatitis [4].

Imaging plays an important role in diagnosis and assessment of complications. B-mode ultrasonography is widely used because it is noninvasive, radiation-free, inexpensive, repeatable, and particularly useful for detecting gallstones and biliary abnormalities [3]. However, pancreatic visualization may be limited by bowel gas and obesity. Contrast-enhanced computed tomography (CECT) provides better assessment of pancreatic necrosis and peripancreatic complications, but involves radiation exposure, contrast-related limitations, and may be normal during the early stage of disease [5]. Magnetic resonance imaging is useful for patients with contrast contraindications and for detailed assessment of the biliary and pancreatic ducts, although its higher cost and longer acquisition time limit routine use [6].

Shear wave elastography (SWE) is a noninvasive, real-time technique that provides quantitative assessment of tissue stiffness. It generates shear waves within tissue and measures their propagation velocity, which increases as tissue becomes stiffer. In acute pancreatitis, inflammatory changes and tissue injury may alter pancreatic mechanical properties, potentially making SWE useful for early diagnosis and severity assessment [7]. SWE has also been investigated in other clinical settings, including musculoskeletal imaging, assessment of adrenal tissue, and evaluation of tissue characteristics in pediatric abdominal conditions [8-10].

Several clinical and radiological scoring systems are available for severity assessment. The Ranson and Acute Physiology and Chronic Health Evaluation II (APACHE II) scores use clinical and biochemical parameters, whereas the Bedside Index for Severity in AP (BISAP) provides a simpler early assessment [11]. Among radiological systems, the Balthazar CT severity index and its modified form incorporate pancreatic inflammation, fluid collections, and necrosis. The modified CT severity index (MCTSI), introduced by Mortele et al., improved prediction of clinically important outcomes including organ dysfunction, intervention, infection, and mortality [12]. The Revised Atlanta Classification (RAC) further categorizes AP into interstitial edematous and necrotizing pancreatitis and provides standardized criteria for severity and complications [3].

This study aimed to evaluate pancreatic acoustic radiation force impulse shear wave elastography (ARFI-SWE) parameters in patients with acute pancreatitis compared with healthy controls and to examine their relationship with MCTSI severity, RAC, and time since symptom onset.

## Materials and methods

Study design and setting

This hospital-based case-control study was conducted in the Department of Radiodiagnosis, Teerthanker Mahaveer Medical College and Research Centre (TMMC & RC), Moradabad, over 18 months, from 1 July 2024 to 31 December 2025, following approval from the Institutional Ethics Committee (TMU/IEC/2024-25/PG/05). The study included patients with acute pancreatitis and healthy controls.

Study population and eligibility

Patients aged >18 years with a clinical diagnosis of acute pancreatitis who were referred for abdominal ultrasonography and provided written informed consent were eligible for inclusion. Patients with chronic pancreatitis, pancreatic malignancy, solid or cystic pancreatic masses, disease confined to the pancreatic tail, previous pancreatic surgery, pregnancy, body mass index (BMI) >30 kg/m², or documented contrast hypersensitivity were excluded. Controls were age- and sex-matched healthy adults without pancreaticobiliary disease and with BMI <30 kg/m² who provided written informed consent.

Sample size

The minimum sample size was calculated for comparison of two proportions using a two-sided significance level of 5%, 80% power, and a 1:1 case-to-control ratio. The sample size per group was calculated using:

$n=\frac{[Z_{\alpha/2}\sqrt{2p(1-p)}+Z_{\beta}\sqrt{p_1(1-p_1)+p_2(1-p_2)}]^2}{(p_1-p_2)^2}$

where is the required sample size in each group, and are the expected proportions in the two groups, for a two-sided α of 0.05, and for 80% power. The calculated minimum sample size was 46 participants per group. The final study population comprised 46 patients with acute pancreatitis and 46 controls.

Ultrasonography and acoustic radiation force impulse (ARFI) elastography

Ultrasonography was performed using a Siemens ACUSON Sequoia ultrasound system with a 6C1 HD convex transducer (Siemens Healthineers, Erlangen, Germany). Participants were examined after fasting in the supine position. B-mode ultrasonography was performed initially to assess pancreatic location, size, echogenicity, morphology, margins, pancreatic duct, and surrounding peripancreatic tissues. Peripancreatic fluid collections and other associated findings were also assessed.

ARFI-based shear wave elastography was performed following B-mode examination using minimal transducer pressure. Participants were instructed to briefly suspend respiration during image acquisition to reduce motion artefacts; however, a specific respiratory phase was not standardized. A 10 × 6 mm region of interest was placed within the pancreatic head and body while avoiding adjacent vessels, liver parenchyma, and bowel. Three measurements were obtained from each region, and the median value was used for analysis. ARFI measurements were recorded as shear wave velocity in m/s and pancreatic stiffness in kPa (Figure 1).

**Figure 1:**
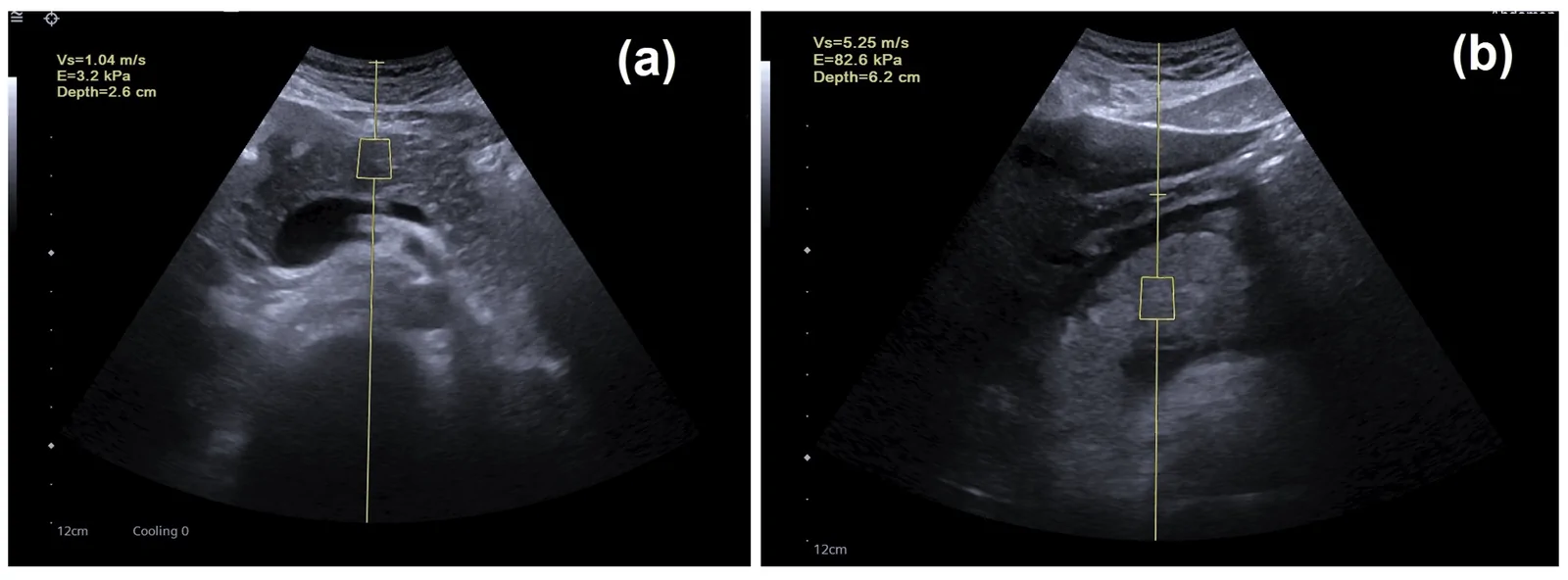
Representative ARFI-SWE images of the pancreas. (a) Healthy control, (b) Patient with acute pancreatitis. ARFI-SWE: acoustic radiation force impulse shear wave elastography.

Computed tomography and MCTSI assessment

Contrast-enhanced CT was performed using a 160-slice UCT780 scanner (United Imaging, Shanghai, China). The examination was performed in the supine position and, when required, in the right lateral decubitus position. The CT protocol used 80-120 kVp, 250-300 mAs, 1-mm slice thickness, and a 2-mm reconstruction increment. Noncontrast and postcontrast examinations of the abdomen and pelvis were obtained. Images were assessed in axial, coronal, and sagittal planes using multiplanar reconstruction, while maximum-intensity-projection images were used for vascular assessment.

The severity of acute pancreatitis was assessed using the MCTSI [12]. For analysis, MCTSI scores were categorized as mild (score 2), moderate (scores 4-6), and severe (scores 8-10). For the severe versus non-severe analysis, scores of 2-6 were classified as non-severe and scores of 8-10 as severe. The RAC was also recorded for each patient and categorized as mild, moderately severe, or severe pancreatitis [3]. The relationship between MCTSI and RAC severity categories was assessed descriptively and using the Fisher-Freeman-Halton exact test.

Clinical and laboratory assessment

Clinical history and relevant laboratory investigations, including serum amylase and lipase, were recorded. Time since symptom onset was categorized as <24 hours, 24-48 hours, and >48 hours.

Statistical analysis

Data were analyzed using IBM SPSS Statistics for Windows, Version 27.0 (IBM Corp., Armonk, NY, USA). Continuous variables were summarized as mean ± SD and categorical variables as n (%). Group comparisons used independent-samples or Welch’s t-test and chi-square/Fisher’s exact test, as appropriate. ARFI parameters across MCTSI severity groups were compared using one-way analysis of variance (ANOVA) with Tukey post hoc testing and confirmed using the Kruskal-Wallis test. Spearman correlation and simple linear regression assessed the relationship between ARFI parameters and MCTSI score. Severe and non-severe groups were compared using Welch’s t-test, and receiver operating characteristic (ROC) analysis assessed discrimination of severe MCTSI-defined pancreatitis. The MCTSI and RAC were compared using the Fisher-Freeman-Halton exact test. A two-sided P<0.05 was considered statistically significant.

## Results

Patients with acute pancreatitis had a higher mean age than controls (44.13 ± 15.71 vs. 38.61 ± 13.21 years), but the difference was not statistically significant (t=1.824, df=90; P=0.071). The sex distribution was also similar between groups, with females comprising 45.7% of patients and 47.8% of controls, while males comprised 54.3% and 52.2%, respectively (χ²=0.044, df=1; P=0.834) (Table 1).

**Table 1:** Baseline demographic characteristics of patients with acute pancreatitis and controls. n: number, SD: standard deviation.

Parameter	Domain	Acute pancreatitis (n=46)	Controls (n=46)	Test statistic	P value
Age (years), mean ± SD		44.13 ± 15.71	38.61 ± 13.21	t=1.824, df=90	0.071
Sex, n (%)	Female	21 (45.7)	22 (47.8)	χ²=0.044, df=1	0.834
	Male	25 (54.3)	24 (52.2)		

Pancreatic head thickness was higher in patients with acute pancreatitis than in controls [30.28 ± 3.81 vs. 25.78 ± 2.85 mm; mean difference 4.500 mm, 95% confidence interval (CI) 3.106-5.894; t=6.413, df=90; P<0.001]. Pancreatic body thickness was also greater in patients (24.35 ± 3.14 vs. 15.24 ± 2.14 mm; mean difference 9.109 mm, 95% CI 7.993-10.225; Welch t=16.245, df=79.378; P<0.001). Main pancreatic duct diameter was higher in patients than in controls (3.226 ± 0.520 vs. 1.243 ± 0.513 mm; mean difference 1.983 mm, 95% CI 1.769-2.197; t=18.404, df=90; P<0.001). ARFI head velocity was higher in patients than controls (2.845 ± 0.476 vs. 1.219 ± 0.143 m/s; mean difference 1.626 m/s, 95% CI 1.479-1.773; Welch t=22.180, df=53.067; P<0.001), as was body velocity (2.949 ± 0.642 vs. 1.161 ± 0.139 m/s; mean difference 1.787 m/s, 95% CI 1.593-1.982; Welch t=18.467, df=49.195; P<0.001). ARFI head stiffness was 22.861 ± 5.947 kPa in patients compared with 4.596 ± 0.731 kPa in controls (mean difference 18.265 kPa, 95% CI 16.487-20.043; Welch t=20.676, df=46.358; P<0.001), while body stiffness was 22.730 ± 6.040 vs. 4.139 ± 0.639 kPa (mean difference 18.591 kPa, 95% CI 16.789-20.394; Welch t=20.761, df=46.008; P<0.001) (Table 2).

**Table 2:** Comparison of pancreatic imaging and ARFI parameters between patients with acute pancreatitis and controls. n: number, SD: standard deviation, CI: confidence interval, ARFI: acoustic radiation force impulse.

Parameter	Acute pancreatitis (n=46), mean ± SD	Controls (n=46), mean ± SD	Test statistic	Mean difference (95% CI)	P value
Pancreatic head thickness (mm)	30.28 ± 3.81	25.78 ± 2.85	t=6.413, df=90	4.500 (3.106–5.894)	<0.001
Pancreatic body thickness (mm)	24.35 ± 3.14	15.24 ± 2.14	Welch t=16.245, df=79.378	9.109 (7.993–10.225)	<0.001
Main pancreatic duct diameter (mm)	3.226 ± 0.520	1.243 ± 0.513	t=18.404, df=90	1.983 (1.769–2.197)	<0.001
ARFI head velocity (m/s)	2.845 ± 0.476	1.219 ± 0.143	Welch t=22.180, df=53.067	1.626 (1.479–1.773)	<0.001
ARFI body shear wave velocity (m/s)	2.949 ± 0.642	1.161 ± 0.139	Welch t=18.467, df=49.195	1.787 (1.593–1.982)	<0.001
ARFI head stiffness (kPa)	22.861 ± 5.947	4.596 ± 0.731	Welch t=20.676, df=46.358	18.265 (16.487–20.043)	<0.001
ARFI body stiffness (kPa)	22.730 ± 6.040	4.139 ± 0.639	Welch t=20.761, df=46.008	18.591 (16.789–20.394)	<0.001

Among the 46 patients with acute pancreatitis, peripancreatic fat stranding/inflammation was the most frequent finding, present in 45 (97.8%) patients, followed by peripancreatic fluid in 33 (71.7%) and interstitial edematous pancreatitis in 31 (67.4%). Ascites was present in 24 (52.2%) patients, while acute peripancreatic fluid collection was seen in 17 (37.0%) and necrotizing pancreatitis in 15 (32.6%). Pancreatic necrosis was present in 13 (28.3%) patients and bilateral pleural effusion in 12 (26.1%). Acute necrotic collection was observed in 11 (23.9%) patients, left pleural effusion in 8 (17.4%), and vascular thrombosis in five (10.9%). Right pleural effusion was not observed in any patient (Table 3).

**Table 3:** Categorical imaging findings among patients with acute pancreatitis (n=46). n = number.

Imaging finding	n (%)
Peripancreatic fat stranding/inflammation	45 (97.8)
Peripancreatic fluid	33 (71.7)
Pancreatic necrosis	13 (28.3)
Vascular thrombosis	5 (10.9)
Thickening of stomach/bowel wall	14 (30.4)
Interstitial edematous pancreatitis	31 (67.4)
Necrotizing pancreatitis	15 (32.6)
Acute peripancreatic fluid collection (<4 weeks)	17 (37.0)
Acute necrotic collection (<4 weeks)	11 (23.9)
Left pleural effusion	8 (17.4)
Right pleural effusion	0 (0.0)
Bilateral pleural effusion	12 (26.1)
Ascites	24 (52.2)

Serum lipase levels among patients with acute pancreatitis were 635.44 ± 169.23, with a 95% CI of 585.18-685.70 and a range of 250.30-1020.00. Serum amylase levels were 1229.00 ± 446.10, with a 95% CI of 1096.52-1361.48 and a range of 147-2250 (Table 4).

**Table 4:** Serum pancreatic enzyme levels among patients with acute pancreatitis (n=46). n: number, SD: standard deviation, CI: confidence interval.

Parameter	n	Mean ± SD	95% CI of mean	Range
Serum lipase	46	635.44 ± 169.23	585.18–685.70	250.30–1020.00
Serum amylase	46	1229.00 ± 446.10	1096.52–1361.48	147.00–2250.00

Among the 46 patients, 15 (32.6%) had mild, 21 (45.7%) had moderate, and 10 (21.7%) had severe pancreatitis according to MCTSI. By the RAC, 13 (28.3%) patients had mild, 22 (47.8%) had moderately severe, and 11 (23.9%) had severe. Moderate disease by MCTSI and moderately severe disease by RAC were the most common categories (Table 5).

**Table 5:** Severity distribution according to MCTSI and RAC among patients with acute pancreatitis (n=46). MCTSI: modified CT severity index, n: number, RAC: Revised Atlanta Classification.

Severity category	MCTSI, n (%)	RAC, n (%)
Mild	15 (32.6)	13 (28.3)
Moderate / Moderately severe	21 (45.7)	22 (47.8)
Severe	10 (21.7)	11 (23.9)
Total	46 (100.0)	46 (100.0)

There was a strong and statistically significant association between MCTSI severity and RAC (Fisher-Freeman-Halton exact test, P<0.001). Most patients classified as mild by MCTSI were also mild by RAC (13/15, 86.7%), while 20 of 21 patients with moderate MCTSI scores (95.2%) were classified as moderately severe by RAC. All patients with severe MCTSI scores were classified as severe by RAC (10/10, 100%) (Table 6).

**Table 6:** Comparison of MCTSI severity category with RAC among patients with acute pancreatitis (n=46). MCTSI: modified CT severity index, RAC: Revised Atlanta Classification, n: number.

MCTSI category	Mild RAC, n (%)	Moderately severe RAC, n (%)	Severe RAC, n (%)	Total	Fisher–Freeman–Halton exact test
Mild	13 (86.7)	2 (13.3)	0 (0.0)	15 (100.0)	P<0.001
Moderate	0 (0.0)	20 (95.2)	1 (4.8)	21 (100.0)	
Severe	0 (0.0)	0 (0.0)	10 (100.0)	10 (100.0)	
Total	13 (28.3)	22 (47.8)	11 (23.9)	46 (100.0)	

ARFI head velocity increased from 2.264 ± 0.255 m/s in mild cases to 3.024 ± 0.232 m/s in moderate and 3.339 ± 0.064 m/s in severe cases (F=86.821, P<0.001). Similarly, body velocity increased from 2.239 ± 0.260 m/s to 3.022 ± 0.237 m/s and 3.857 ± 0.246 m/s across mild, moderate, and severe categories, respectively (F=130.882, P<0.001). ARFI head stiffness increased from 15.573 ± 3.472 kPa in mild cases to 25.538 ± 2.958 kPa in moderate and 28.170 ± 1.441 kPa in severe cases (F=72.901, P<0.001). Body stiffness showed a similar increase from 15.260 ± 3.497 kPa to 25.505 ± 2.875 kPa and 28.110 ± 1.371 kPa, respectively (F=78.373, P<0.001) (Table 7).

**Table 7:** ARFI parameters according to MCTSI severity. ARFI: acoustic radiation force impulse, SD: standard deviation, F: F statistic.

ARFI parameter	Mild (score 2), mean ± SD	Moderate (scores 4–6), mean ± SD	Severe (scores 8–10), mean ± SD	F (2,43)	P value
ARFI head velocity (m/s)	2.264 ± 0.255	3.024 ± 0.232	3.339 ± 0.064	86.821	<0.001
ARFI body shear wave velocity (m/s)	2.239 ± 0.260	3.022 ± 0.237	3.857 ± 0.246	130.882	<0.001
ARFI head stiffness (kPa)	15.573 ± 3.472	25.538 ± 2.958	28.170 ± 1.441	72.901	<0.001
ARFI body stiffness (kPa)	15.260 ± 3.497	25.505 ± 2.875	28.110 ± 1.371	78.373	<0.001

MCTSI score showed a strong positive correlation with all ARFI parameters. The strongest correlation was observed with ARFI body velocity (Spearman ρ=0.898, P<0.001), followed by ARFI head velocity (ρ=0.839, P<0.001), body stiffness (ρ=0.811, P<0.001), and head stiffness (ρ=0.805, P<0.001) (Table 8).

**Table 8:** Correlation of MCTSI score with ARFI parameters. ARFI: acoustic radiation force impulse, MCTSI: modified CT severity index.

ARFI parameter	Spearman ρ	P value
ARFI head velocity	0.839	<0.001
ARFI body velocity	0.898	<0.001
ARFI head stiffness	0.805	<0.001
ARFI body stiffness	0.811	<0.001

In simple linear regression, all four ARFI parameters showed a significant positive association with MCTSI score. ARFI body velocity had the highest explanatory value, with R=0.887, R²=0.786, and adjusted R²=0.781; its regression coefficient was B=3.701 (95% CI 3.114-4.288), t=12.714, P<0.001. ARFI body stiffness showed R=0.823, R²=0.677, and adjusted R²=0.670, with B=0.365 (95% CI 0.288-0.442), t=9.605, P<0.001. ARFI head stiffness showed R=0.821, R²=0.674, and adjusted R²=0.667, with B=0.370 (95% CI 0.292-0.448), t=9.537, P<0.001. ARFI head velocity showed R=0.840, R²=0.706, and adjusted R²=0.699, with B=4.726 (95% CI 3.799-5.653), t=10.275, P<0.001 (Table 9).

**Table 9:** Simple linear regression models for prediction of MCTSI score. R: multiple correlation coefficient, B: unstandardized regression coefficient, CI: confidence interval, ARFI: acoustic radiation force impulse.

Predictor	R	R²	Adjusted R²	B (95% CI)	t	P value
ARFI head velocity	0.840	0.706	0.699	4.726 (3.799–5.653)	10.275	<0.001
ARFI body velocity	0.887	0.786	0.781	3.701 (3.114–4.288)	12.714	<0.001
ARFI head stiffness	0.821	0.674	0.667	0.370 (0.292–0.448)	9.537	<0.001
ARFI body stiffness	0.823	0.677	0.670	0.365 (0.288–0.442)	9.605	<0.001

ARFI measurements were significantly higher in patients with severe pancreatitis than in those with non-severe pancreatitis. ARFI head velocity was 3.339 ± 0.064 m/s in severe cases compared with 2.708 ± 0.449 m/s in non-severe cases (Welch t=8.154; 95% CI −0.788 to −0.475 for the non-severe minus severe difference; P<0.001). Body velocity was 3.857 ± 0.246 vs. 2.696 ± 0.461 m/s (Welch t=10.613; 95% CI −1.385 to −0.937; P<0.001). Head stiffness was 28.170 ± 1.441 vs. 21.386 ± 5.886 kPa (Welch t=6.272; 95% CI −8.964 to −4.604; P<0.001), while body stiffness was 28.110 ± 1.371 vs. 21.236 ± 5.988 kPa (Welch t=6.318; 95% CI −9.067 to −4.680; P<0.001) (Table 10).

**Table 10:** ARFI parameters according to severe versus non-severe MCTSI classification. SD: standard deviation, CI: confidence interval, ARFI: acoustic radiation force impulse, MCTSI: modified CT severity index.

Parameter	Non-severe (n=36), mean ± SD	Severe (n=10), mean ± SD	Welch t	95% CI of mean difference*	P value
ARFI head velocity (m/s)	2.708 ± 0.449	3.339 ± 0.064	−8.154	−0.788 to −0.475	<0.001
ARFI body shear wave velocity (m/s)	2.696 ± 0.461	3.857 ± 0.246	−10.613	−1.385 to −0.937	<0.001
ARFI head stiffness (kPa)	21.386 ± 5.886	28.170 ± 1.441	−6.272	−8.964 to −4.604	<0.001
ARFI body stiffness (kPa)	21.236 ± 5.988	28.110 ± 1.371	−6.318	−9.067 to −4.680	<0.001

ARFI showed good diagnostic performance for identifying severe pancreatitis according to MCTSI. ARFI head velocity had an area under the curve (AUC) of 0.935 (95% CI 0.862-1.000; P<0.001). ARFI body velocity showed the highest discrimination, with an AUC of 1.000 (95% CI 1.000-1.000; P<0.001). ARFI head stiffness had an AUC of 0.854 (95% CI 0.748-0.961; P=0.001), while body stiffness had an AUC of 0.858 (95% CI 0.753-0.963; P=0.001) (Table 11, Figure 2).

**Table 11:** Diagnostic performance of ARFI for severe MCTSI-defined pancreatitis. AUC: area under the curve, CI: confidence interval, ARFI: acoustic radiation force impulse.

ARFI parameter	AUC	95% CI	P value
ARFI head velocity	0.935	0.862–1.000	<0.001
ARFI body velocity	1.000	1.000–1.000	<0.001
ARFI head stiffness	0.854	0.748–0.961	0.001
ARFI body stiffness	0.858	0.753–0.963	0.001

**Figure 2:**
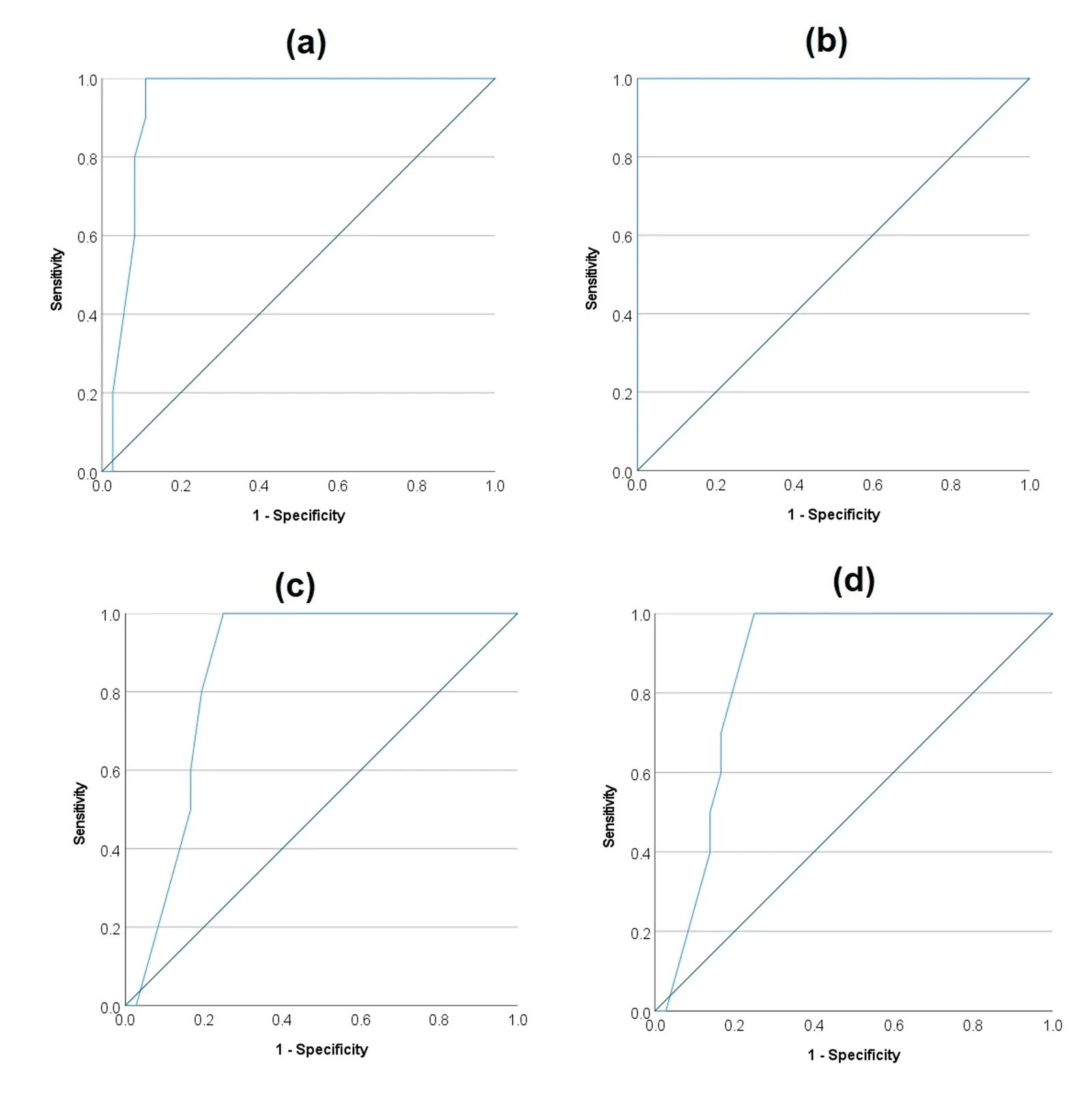
ROC curves for ARFI parameters in the prediction of severe acute pancreatitis according to the MCTSI. (a) ARFI head velocity; (b) ARFI body velocity; (c) ARFI head stiffness; and (d) ARFI body stiffness. ROC: receiver operating characteristic, ARFI: acoustic radiation force impulse, MCTSI: modified CT severity index.

ARFI measurements increased significantly with increasing time since symptom onset. Patients presenting within <24 hours had a head velocity of 2.186 ± 0.311 m/s, body velocity of 2.167 ± 0.324 m/s, head stiffness of 14.589 ± 4.333 kPa, and body stiffness of 14.378 ± 4.521 kPa. These values increased to 2.926 ± 0.382 m/s, 3.053 ± 0.596 m/s, 24.314 ± 4.842 kPa, and 24.200 ± 4.931 kPa, respectively, at 24-48 hours, and to 3.109 ± 0.299 m/s, 3.251 ± 0.475 m/s, 25.606 ± 3.573 kPa, and 25.500 ± 3.612 kPa after >48 hours. The differences across the three time groups were significant for all four parameters (F=22.039, 13.657, 20.707, and 20.240, respectively; all P<0.001) (Table 12).

**Table 12:** ARFI parameters according to time since symptom onset. n: number, ARFI: acoustic radiation force impulse.

ARFI parameter	<24 hours (n=9)	24–48 hours (n=21)	>48 hours (n=16)	Test statistic	P value
ARFI head velocity (m/s)	2.186 ± 0.311	2.926 ± 0.382	3.109 ± 0.299	F=22.039	<0.001
ARFI body shear wave velocity (m/s)	2.167 ± 0.324	3.053 ± 0.596	3.251 ± 0.475	F=13.657	<0.001
ARFI head stiffness (kPa)	14.589 ± 4.333	24.314 ± 4.842	25.606 ± 3.573	F=20.707	<0.001
ARFI body stiffness (kPa)	14.378 ± 4.521	24.200 ± 4.931	25.500 ± 3.612	F=20.240	<0.001

## Discussion

The present study evaluated pancreatic morphology and ARFI shear wave elastography in 46 patients with acute pancreatitis and 46 healthy controls. Patients with acute pancreatitis had significantly greater pancreatic head and body thickness, main pancreatic duct diameter, shear wave velocity, and pancreatic stiffness than controls. ARFI parameters also showed strong associations with MCTSI severity and increased with longer duration of symptoms, supporting their potential value as noninvasive markers of disease severity.

Pancreatic head thickness was significantly higher in patients with acute pancreatitis than in controls (30.28 ± 3.81 vs. 25.78 ± 2.85 mm), while body thickness was also increased (24.35 ± 3.14 vs. 15.24 ± 2.14 mm). These findings indicate pancreatic enlargement associated with acute inflammation and edema. The results are comparable with Brizi et al. [5], who reported pancreatic head measurements of approximately 30-34 mm in acute pancreatitis. O’Connor et al. [6] reported values of approximately 28-32 mm, while Sun et al. [13] described body measurements in the range of 24-28 mm. Grassedonio et al. [14] also reported increased pancreatic dimensions in acute pancreatitis. Thus, the present findings are broadly consistent with previous imaging studies.

The main pancreatic duct was also significantly wider in patients than in controls (3.226 ± 0.520 vs. 1.243 ± 0.513 mm). Similar values have been reported by Kurihara et al. [15], O’Connor et al. [6], and Sun et al. [13]. The increased duct diameter may reflect inflammatory edema, transient obstruction, or impaired pancreatic drainage. The considerably smaller diameter in controls is consistent with normal pancreatic duct anatomy.

The present study demonstrated significantly higher ARFI velocities in acute pancreatitis. Mean head velocity was 2.845 ± 0.476 m/s compared with 1.219 ± 0.143 m/s in controls, while body velocity was 2.949 ± 0.642 m/s compared with 1.161 ± 0.139 m/s. These findings are comparable with Goertz et al. [7], who reported velocities of approximately 2.5-3.0 m/s in acute pancreatitis. Durmaz et al. [16], Kaya et al. [17], and Basavaraju et al. [18] also reported elevated shear wave velocities in patients with pancreatitis.

The increased velocity probably reflects the mechanical changes produced by pancreatic inflammation and edema. The substantially lower velocities in controls provide further evidence that ARFI can distinguish altered pancreatic tissue from normal pancreatic parenchyma.

A marked difference was also observed in stiffness measurements. Head stiffness was 22.861 ± 5.947 kPa in patients compared with 4.596 ± 0.731 kPa in controls, while body stiffness was 22.730 ± 6.040 versus 4.139 ± 0.639 kPa. These findings are broadly comparable with reports from Kawada et al. [19], Xie et al. [20], Sezgin et al. [21], and Hristov et al. [22], who described substantially higher pancreatic stiffness in acute pancreatitis than in controls.

Importantly, ARFI measurements increased progressively across MCTSI severity categories. Head velocity increased from 2.264 ± 0.255 m/s in mild disease to 3.024 ± 0.232 m/s in moderate and 3.339 ± 0.064 m/s in severe disease. Similar increases were observed for body velocity and both head and body stiffness, with all comparisons being statistically significant. This supports the relationship between pancreatic mechanical properties and radiological severity. Previous studies by Goertz et al. [7], Durmaz et al. [16], Kaya et al. [17], and Basavaraju et al. [18] also reported significant associations between elastography measurements and severity indices.

The correlation analysis further supported this relationship. MCTSI score showed strong positive correlations with head velocity (ρ=0.839), body velocity (ρ=0.898), head stiffness (ρ=0.805), and body stiffness (ρ=0.811), with all P<0.001. Among the parameters, body velocity showed the strongest correlation. Simple linear regression similarly demonstrated significant positive associations for all four ARFI measures, with body velocity showing the highest explanatory value (R²=0.786). These findings are consistent with previous work showing that elastography parameters can reflect increasing disease severity [7, 16, 17, 20].

ARFI measurements were significantly higher in severe than in non-severe pancreatitis. The ROC analysis showed good to excellent discrimination for severe MCTSI-defined disease. Head velocity had an AUC of 0.935, body velocity an AUC of 1.000, while head and body stiffness had AUCs of 0.854 and 0.858, respectively. These findings compare favorably with previous studies reporting AUC values in the range of approximately 0.82-0.90 for severity assessment [7, 16, 17, 20]. The particularly high AUC for body velocity in the present dataset should, however, be interpreted cautiously because the severe group contained only 10 patients.

ARFI values also increased significantly with increasing time since symptom onset. Head velocity increased from 2.186 ± 0.311 m/s within <24 hours to 2.926 ± 0.382 m/s at 24-48 hours and 3.109 ± 0.299 m/s after >48 hours. Similar increases were observed for body velocity and both stiffness measurements. The observed pattern may reflect differences in pancreatic inflammatory changes across patients presenting at different times after symptom onset. Comparable observations have been reported by Kaya et al. [17], Sezgin et al. [21], Durmaz et al. [16], Xie et al. [20], Basavaraju et al. [18], and Makhlouf et al. [23]. Because the symptom-duration groups consisted of different patients rather than serial measurements in the same individuals, these findings should be interpreted as an association between symptom duration and ARFI measurements rather than evidence of temporal progression within individual patients.

In the present study, there was a strong agreement between MCTSI-based severity assessment and the RAC. Most patients categorized as mild by MCTSI were also classified as mild by RAC, while the majority of patients with moderate MCTSI scores were classified as moderately severe by RAC. Importantly, all patients with severe MCTSI scores were classified as severe according to RAC. The significant association between the two classifications (Fisher-Freeman-Halton exact test=64.516, P<0.001) supports the consistency of MCTSI-based severity assessment with the RAC. This finding also provides clinical context for the observed association between ARFI parameters and MCTSI severity.

This study has some limitations. It was conducted at a single tertiary care centre with a relatively small sample size, which may limit the generalizability of the findings. The cross-sectional assessment of ARFI measurements limits conclusions about changes in pancreatic stiffness over the longer course of disease. Only a limited number of patients had severe pancreatitis, which may affect the precision of ROC estimates. In addition, ARFI measurements can be influenced by technical factors such as bowel gas, patient habitus, respiratory motion, and transducer pressure. Formal intraobserver and interobserver reproducibility of ARFI-SWE measurements was not assessed, which may limit assessment of measurement variability. The interval between ARFI-SWE and CT was not standardized/documented, which may have introduced variability because pancreatic inflammatory and morphological findings can change over time. Respiratory motion and respiratory phase may also influence pancreatic SWE measurements, and previous work has examined differences in pancreatic stiffness measurements between breath-hold and free-breathing conditions [24]. Larger multicentre prospective studies with external validation are needed to confirm these findings.

## Conclusions

ARFI shear wave elastography demonstrated significantly higher pancreatic shear wave velocity and stiffness in patients with acute pancreatitis than in healthy controls. ARFI parameters increased progressively with increasing MCTSI severity and showed strong positive correlations with MCTSI score, with body velocity demonstrating the strongest correlation and highest explanatory value. All four ARFI parameters showed good to excellent discrimination of severe MCTSI-defined pancreatitis. ARFI measurements also increased significantly with longer symptom duration. These findings suggest that ARFI-SWE may be a useful, noninvasive quantitative adjunct to conventional imaging for assessing pancreatic involvement and severity in acute pancreatitis.
